# Sexual Satisfaction, Performance, and Partner Response Following Voluntary Medical Male Circumcision in Zambia: The Spear and Shield Project

**DOI:** 10.9745/GHSP-D-15-00163

**Published:** 2015-12-15

**Authors:** Robert Zulu, Deborah Jones, Ndashi Chitalu, Ryan Cook, Stephen Weiss

**Affiliations:** ^a^​ University of Zambia School of Medicine, Lusaka, Zambia; ^b^​ University of Miami Miller School of Medicine, Psychiatry and Behavioral Sciences, Miami, FL, USA

## Abstract

**Background:**

Voluntary medical male circumcision (VMMC) is an important HIV prevention strategy, particularly in regions with high HIV incidence and low rates of male circumcision. However, 88% of the Zambian male population remain uncircumcised, and of these 80% of men surveyed expressed little interest in undergoing VMMC.

**Methods:**

The Spear and Shield study (consisting of 4 weekly, 90-minute sexual risk reduction/VMMC promotion sessions) recruited and enrolled men (N = 800) who self-identified as at risk of HIV by seeking HIV testing and counseling at community health centers. Eligible men tested HIV-negative, were uncircumcised, and expressed no interest in VMMC. Participants were encouraged (but not required) to invite their female partners (N = 668) to participate in the program in a gender-concordant intervention matched to their partners’. Men completed assessments at baseline, post-intervention (about 2 months after baseline), and 6 and 12 months post-intervention; women completed assessments at baseline and post-intervention. For those men who underwent VMMC and for their partners, an additional assessment was conducted 3 months following the VMMC. The ancillary analysis in this article compared the pre- and post-VMMC responses of the 257 Zambian men who underwent circumcision during or following study participation, using growth curve analyses, as well as of the 159 female partners.

**Results:**

Men were satisfied overall with the procedure (mean satisfaction score, 8.4 out of 10), and nearly all men (96%) and women (94%) stated they would recommend VMMC to others. Approximately half of the men reported an increase or no change in erections, orgasms, and time to achieve orgasms from pre-VMMC, while one-third indicated fewer erections and orgasms and decreased time to achieve orgasms post-VMMC. Nearly half (42%) of the men, and a greater proportion (63%) of the female partners, said their sexual pleasure increased while 22% of the men reported less sexual pleasure post-VMMC. Growth curve analysis of changes in sexual functioning and satisfaction over time revealed no changes in erectile functioning or intercourse satisfaction, but there were increases in orgasm functioning, overall sexual satisfaction, and sexual desire. The majority (61% to 70%) of men and women thought penile cleanliness and appearance had improved post-VMMC. Of the 69% of men who reported having sexual intercourse at least once between having the procedure and their 3-month post-VMMC assessment, the large majority (76%) waited at least 6 weeks before resuming sex. Sexual intercourse prior to the 6-week healing period was associated with adverse events and lower levels of post-VMMC sexual satisfaction.

**Conclusion:**

Both men and their partners can generally expect equal or improved sexual satisfaction and penile hygiene following VMMC. Future studies should consider innovative strategies to assist men in their efforts to abstain from sexual activities prior to complete healing.

## Abstract (precis)

Most men and their partners reported increased or the same levels of sexual pleasure and improved or no change in penile hygiene post-VMMC. While half of men reported increased or no change in sexual functioning (orgasm, erections), one-third reported a decrease. Early resumption of sexual intercourse prior to complete healing was most closely associated with adverse outcomes, including decreased sexual functioning, satisfaction, and desire.

## INTRODUCTION

Male circumcision has been practiced for nearly 4,500 years for religious, cultural, and medical reasons. It has also stimulated controversy among religious, cultural, and medical authorities, particularly with regard to its relevance in modern society.^1^ Voluntary medical male circumcision (VMMC), however, has recently been recognized as an important barrier to HIV infection in men (51% to 70% reduction in risk), and it has been recommended as an important HIV prevention strategy particularly in countries or regions with high HIV incidence and low rates of male circumcision, e.g., Eastern and Southern Africa.^2^^–^^4^ Lower rates of HIV infection resulting from medical circumcision in men would also reduce the risk of exposure to HIV infection among women, ultimately conveying an estimated 46% reduction in women’s risk of infection.^5^

Voluntary medical male circumcision (VMMC) has been shown to reduce HIV acquisition risk in men by 51% to 70%.

Despite these impressive statistics, the Zambia Sexual Behaviour Survey conducted in 2009^6^ found that as many as 80% of uncircumcised Zambian men (i.e., 88% of the male population) expressed little interest in undergoing VMMC. Studies exploring reasons for men’s unwillingness to be circumcised have identified concerns related to potential effects of VMMC on sexual performance (e.g., erection, orgasm) and sexual pleasure, the risk of surgical pain, reluctance to abstain from sex for at least 6 weeks during recovery, and partners’ responses to the loss of the penile foreskin.^7^^,^^8^^–^^10^ Studies in Kenya, Uganda, South Africa, and Zambia have found the majority of men who had undergone VMMC were satisfied with the procedure.^8^^,^^11^^–^^14^ Further, studies in Kenya, Malawi, South Africa, and Tanzania have found that women report high sexual satisfaction with circumcised partners and believe that circumcision improves appearance, health, and hygiene.^8^^,^^12^^,^^15^^,^^16^ Additionally, research has found that female partners can influence men’s uptake of VMMC.^13^^,^^17^^,^^18^ However, no studies have examined the sexual satisfaction of Zambian women following their partners’ VMMC, nor their perceptions of post-VMMC penile hygiene or appearance.

The large majority of men in Zambia are uncircumcised and have little interest in the procedure.

The Spear and Shield project was a comprehensive sexual risk reduction and VMMC promotion intervention designed to promote VMMC for men initially uninterested in the procedure.^19^ This article examines the post-VMMC experience, including sexual satisfaction and performance, of the male study participants and their female partners. Given the controversies surrounding male circumcision and the disagreements concerning its effect on sexual performance and satisfaction for men and women,^20^^,^^21^ these data could offer definitive guidance to those considering undergoing the procedure, providing valuable information for HIV prevention strategies and implementation of VMMC programs.

## METHODS

### Study Procedures

The ancillary analysis presented in this article was conducted as a component of the Spear and Shield study, a prospective cluster-randomized 3-arm trial conducted in Lusaka, Zambia, between February 1, 2012 and October 31, 2014, to increase uptake of VMMC among Zambian men who were initially uninterested in undergoing the procedure. Thirteen Zambian community health centers (CHCs) were stratified according to size and volume of clients coming for HIV voluntary testing and counseling (HTC) and randomized to the experimental condition (5 clinics), the control condition (5 clinics), or the “observation-only” condition (3 clinics). Men attending HTC at the CHCs were enrolled in the study (N = 800; experimental, n = 400; control, n = 400) and given the option of inviting their female partners to participate (N = 668); no participants were recruited from observation-only sites. Men and women attended parallel group interventions either promoting VMMC for HIV prevention or a time-equivalent control condition.

The primary outcome measure for the study was VMMC uptake; of the 800 men enrolled, 257 men (experimental condition, n = 161; control condition, n = 96) underwent VMMC during the course of the study.^19^^,^^22^ Secondary outcomes included maintenance of sexual barrier use following VMMC,^19^ the acceptability of VMMC,^22^ female partner influence on VMMC uptake,^13^ and sexual satisfaction following VMMC. The analysis in this article focuses on sexual satisfaction and post-VMMC experiences of the 257 male study participants who underwent VMMC and the 159 female partners who completed post-VMMC questionnaires.

### Study Sites and Participants

Three health care providers (nurses or clinic officers) from each of the 13 participating CHC clinics were trained over 10 days to perform male circumcisions using the dorsal slit method, according to VMMC training guides from the World Health Organization.^23^ VMMC training and supplies were provided to all clinics to ensure that clients from all 13 sites had equal access to VMMC services. Two HTC counselors and/or nurses from each of the 10 experimental and control sites were trained to conduct either the Spear and Shield sexual risk reduction program (experimental condition: 4 weekly, 90-minute sexual risk reduction/VMMC promotion sessions) or a time-equivalent, video-based health educational program on malaria, tuberculosis, and waterborne disease prevention (control condition). The experimental and control clinics each recruited a total of 8–9 cohorts of 8–10 male participants, for a total of 80 male participants per site over the course of the study. Observation-only clinics did not recruit any participants or conduct assessments, but they collected monthly data on numbers of HTCs and VMMCs to measure secular trends in VMMC uptake over the course of the study. At recruitment onset, all clinics had trained staff available offering VMMC using the conventional dorsal slit method. (The minimally surgical Shang Ring method and the nonsurgical PrePex method were not available in Zambia at the time of this study.)

Screening, recruitment, and referral of study candidates were carried out by site HTC counselors as part of their post-test counseling activities. Since all candidates were voluntarily seeking HIV testing and counseling, and thus self-identified as at risk of HIV, the candidates were routinely advised of the availability of VMMC services for HIV prevention. Study recruitment targeted the approximately 80% of men identified by the 2009 Zambia Behavioural Health Survey as uninterested in VMMC.

Study assessors were notified by HTC counselors of potential study candidates; study assessors then obtained informed consent from eligible candidates and supervised assessments. Men and women provided consent individually in a private room in the study offices. Eligible male participants were 18 years of age or older, were not infected with HIV, were uncircumcised, and had no plans to undergo VMMC in the foreseeable future. Men were excluded if they were actively considering undergoing VMMC, were infected with HIV (WHO does not recommend circumcision for men with HIV^24^^,^^25^), or had a complicating medical condition preventing VMMC.

All men were encouraged, but not required, to invite their female partners to enroll in a comparable VMMC promotional (or time-equivalent control) program for women. There were no recruitment or screening criteria for female partners, and only those invited by the enrolled men and who had consented to participate were enrolled. All participants were provided with refreshments and compensated for transportation to the site for each visit (about US$4/visit).

### Intervention

Following enrollment, all participants completed a baseline assessment (see next section on Assessments) and then participated in either the experimental or control condition. The experimental condition—the Spear and Shield intervention—consisted of 4 weekly, 90-minute, manual-based, gender-concordant group sessions delivered to 8–10 participants per group. Examples of topics covered in the sessions included HIV prevention strategies, safer sex training in the correct use of male and female condoms, myths and facts about transmission of HIV and sexually transmitted infections (STIs), prevention of mother-to-child transmission, and cognitive behavioral training to improve problem solving in sexual communication and negotiation skills, including identifying and avoiding high-risk sexual situations. VMMC was highlighted in all sessions, which included a detailed description of risks and benefits of VMMC, visits from a peer who had undergone VMMC, and consultation with a VMMC provider who discussed the procedure and recovery. VMMC was discussed in the context of overall sexual risk reduction (e.g., use of condoms, reduction in sexual partners, and avoidance of high-risk environments involving sex, alcohol, and drugs). Men’s and women’s groups followed parallel topics, including gender-specific issues, for example, intimate partner violence and effects of VMMC on women’s health. The control condition was a time-equivalent, video-based health educational program on malaria, tuberculosis, and water-borne disease prevention. Both experimental and control conditions were delivered in local languages. Additional details on the Spear and Shield intervention have been published elsewhere.^13^^,^^14^

### Assessments

All men in the experimental and control conditions completed questionnaires at 4 fixed time points: baseline, post-intervention (about 2 months after baseline), 6 months post-intervention, and 12 months post-intervention. Female partners completed assessments at baseline and post-intervention. For those men who underwent VMMC and for their partners, if enrolled, an additional assessment was conducted at 3 months following the VMMC. All questionnaires were provided in local languages (Bemba, English, or Nyanja), and all assessment data were collected using automated computer-assisted self-interviews (ACASI) in order to minimize social desirability bias. Participants were instructed how to use the ACASI system prior to administration, and a staff member was available to answer questions throughout the assessment.

#### Content of the Questionnaires

**Demographics.** Men and women completed a demographic questionnaire at baseline.

**Sexual functioning and satisfaction.** Men were assessed at all time points on sexual functioning and satisfaction using the International Index of Erectile Functioning (IIEF).^26^ The IIEF yields 5 subscales. The first 2, *erectile functioning* (the ability to achieve/maintain erections during sexual intercourse) and *intercourse satisfaction* (satisfaction with sexual intercourse) are rated 0 (no sexual intercourse), 1 (low), or up to 5 (high). Participants not engaging in sexual intercourse were excluded from these subscales. *Orgasm functioning* (the ability to achieve orgasm during sexual activity including intercourse, oral sex, or masturbation) is also measured from 0 (no sexual intercourse), 1 (low), or up to 5 (high); however, participants reporting no sexual intercourse were not excluded from this subscale, as other types of sexual stimulation are also measured. *Overall satisfaction* (satisfaction with sexual activity in general) and *sexual desire* are rated using a scale of 1 (low) to 5 (high).

Men’s sexual functioning and satisfaction post-VMMC were assessed using the International Index of Erectile Functioning.

**Sexual risk behaviors.** Men and women were also assessed on sexual risk behaviors over the last month at all time points.^27^ Among men, baseline sexual risk behaviors were combined to identify participants at “high risk” and “low risk” for HIV using latent class analysis; indicators of risk category included lifetime STI diagnosis, condom use, multiple partnering, use of drugs or alcohol before sex, and sex with a discordant partner or partner with an unknown HIV status. The analysis has been previously described, and the same risk groups have been used in this study.^28^

**VMMC knowledge.** VMMC knowledge was assessed among men and women at all time points using a measure adapted from a study in Uganda.^12^ Participants answered questions about the ability of VMMC to reduce their HIV risk (e.g., circumcision of a man without HIV reduces his chance of getting HIV; 1 = definitely false, 5 = definitely true) or to completely negate the risk of getting HIV (e.g., a circumcised man cannot get HIV; 1 = definitely false, 5 = definitely true). Items were combined into separate scales representing VMMC knowledge (4-item α = .58) and VMMC misinformation (2-item α = .70).^29^ Both scales were coded such that higher scores indicated more knowledge or misinformation.

**Post-VMMC questionnaire.** At 3 months post-VMMC, men completed an additional questionnaire addressing sexual function and satisfaction, the VMMC experience (including self-reported problems arising post-VMMC, i.e., infection, tearing, healing, pain), and resumption of sexual intercourse following VMMC. Participants were asked to evaluate their experience with VMMC, to indicate the length of time they waited prior to resuming sexual intercourse (if they had intercourse), and to report any problems or complications arising from the procedure. In addition, participants responded to descriptors associated with themselves as circumcised men, using a dichotomous response (1 = yes, 0 = no) to items including satisfaction, appearance, cleanliness, response from partner, and recommendation of VMMC to a friend. Women participating in the study completed a similar questionnaire following their partner’s VMMC; however, women were not assessed regarding complications, sexual function, or delaying intercourse.

### Ethical Oversight

Prior to study initiation, ethical approval was obtained by the University of Zambia Research Ethics Committee and the Institutional Review Board at the University of Miami Miller School of Medicine. The Spear and Shield trial protocol is registered on Clinicaltrials.gov, number NCT01688167.

### Statistical Analyses

Prior to the primary analyses, descriptive statistics (e.g., means, standard deviations, frequencies) were generated for demographic and VMMC satisfaction data. The primary analyses were a series of growth curves examining longitudinal mean subscale scores from the IIEF among men undergoing VMMC. Growth models are statistical models that can be used with repeated-measures data to understand the process of change; they attempt to estimate between-person differences in within-person change. (See Curran et al.^30^ for a concise, nontechnical overview of the growth curve modeling.) The growth models generated for this study included 5 measurement occasions (baseline, post-intervention, 6-month follow-up, 12-month follow-up, post-VMMC) and accounted for the fact that participants completed the post-VMMC assessment at different times relative to baseline. Models included fixed and random intercept, linear, and quadratic time parameters; if the fixed quadratic parameter was not significant, quadratic terms were dropped from the model. In addition, models controlled for the number of attempts at sexual activity at each time point.

In addition to growth curve analyses, descriptive data from the 3-month post-VMMC assessment are presented for both men and their female partners, and Kappa coefficients were computed to examine agreement between partners on questions that were answered by both men and women. Finally, participants who waited the full 6-week healing period prior to resuming sexual intercourse were compared with those who did not wait, using *t* tests, the Wilcoxon signed rank test, and chi-square tests. All analyses were completed using SAS version 9.3 at a 2-tailed level of significance of *P*<.05.

## RESULTS

### Demographics and Satisfaction With the VMMC Procedure

A total of 977 men were screened for the broader Spear and Shield study; 800 men consented to be in the study and were enrolled. Of the 177 men who were not enrolled, 61 declined due to work or distance from the clinic, 28 were under age 18, 12 had planned to undergo VMMC, 18 were unwilling to participate, 7 were previously circumcised, 9 refused to consent, and 42 were disqualified because of their HIV serostatus. A total of 668 women were invited and elected to participate in the study. Additional details on participant recruitment and retention have been published elsewhere.^14^

Male participants undergoing circumcision (N = 257) from the intervention and control arms were, on average, 26 years of age, and nearly three-quarters (74%) had at least 12 years of formal education (Table 1). Over half (57%) were unemployed, and 53% had an annual income of less than US$100. Over one-third (38%) reported being married or living with their partner, about one-third (34%) had at least 1 child, and two-fifths (40%) expressed desire for additional children. Female partners (N = 159) were similar in age (mean, 26 years), and 57% had at least 12 years of education. Most (72%) of the women were unemployed, and 57% reported an annual income of less than US$100. A higher percentage of women than men reported being married or cohabitating (47%), which is not uncommon in Zambia, where men and women often differ on the definition of marriage. Half of the women (52%) had at least 1 child, and 44% reported desire for at least 1 more child.

**TABLE 1:** Demographic Characteristics of Zambian Men Undergoing Voluntary Medical Male Circumcision (N = 257) and Female Partners (N = 159) (Control and Intervention Arms)

Characteristic	Men	Women
Age, mean (SD), years	26.4 (8.2)	26.1 (8.0)
Employment status, No. (%)		
Employed	111 (43%)	45 (28%)
Unemployed	146 (57%)	114 (72%)
Annual income,^a^ No. (%)		
≥ US $100	120 (47%)	69 (43%)
< US $100	137 (53%)	90 (57%)
Education level, No. (%)		
≥ 12 years of education	190 (74%)	90 (57%)
< 12 years of education	67 (26%)	69 (43%)
Relationship status, No. (%)		
Married or cohabitating	97 (38%)	74 (47%)
Not married/Not living with partner	160 (62%)	85 (53%)
Children, No. (%)		
At least one child	88 (34%)	82 (52%)
No children	169 (66%)	77 (48%)
Desire for (more) children, No. (%)		
Yes	103 (40%)	70 (44%)
No	154 (60%)	89 (56%)

Abbreviation: SD, standard deviation.

### Sexual Functioning

Subscales of the IIEF were analyzed using longitudinal growth curves (Table 2). Domains of self-reported sexual functioning analyzed included erectile functioning and orgasm functioning. Examination of erectile functioning scores revealed that both the quadratic and linear time components of the growth curve were not significantly different from zero (*P* values of .08), and thus erectile functioning did not change over time. However, analysis of orgasm functioning scores over time showed a significant linear increase (*P*<.001) and a significant quadratic decrease (*P* = .006) over time, such that the predicted mean scores were 4.12, 4.81, and 4.99 at baseline, 6 months, and 12 months, respectively. Thus, orgasm functioning increased, but the rate of increase diminished over time (Table 2).

Erectile function did not change post-VMCC, but orgasm function increased.

**TABLE 2:** Growth Curve Analyses of International Index of Erectile Functioning Subscales Among Zambian Men Undergoing Voluntary Medical Male Circumcision

Domain	Quadratic Time^a^ b (SE)	*P*	Linear Time b (SE)	*P*	Baseline Mean Estimate^b^	6-Month Mean Estimate	12-Month Mean Estimate
Erectile functioning	-.007 (.004)	.08	.068 (.039)	.08			
Orgasm functioning	**-.007 (.002)**	**.006**	**.156 (.044)**	**<.001**	4.12	4.81	4.99
Intercourse satisfaction	.004 (.002)	.09	.003 (.017)	.84			
Overall satisfaction	-.002 (.003)	.42	**.037 (.013)**	**.007**	7.09	7.31	7.53
Sexual desire	**-.004 (.002)**	**.02**	**.093 (.028)**	**.001**	6.25	6.67	6.79

Abbreviation: SE, standard error.

a All models initially included both quadratic and linear growth curve components (i.e., fixed and random effects for time^2^ and time); if the quadratic component was not significant, the model was refit using only a linear slope.

b Means were estimated using the fitted model for domains that significantly changed over time.

Note: Statistically significant parameters are noted in boldface.

Data from participant assessments 3 months after their VMMC are summarized in Table 3. (One male participant was lost to follow-up following VMMC, thus the valid sample size for post-VMMC data is 256 men.) At post-VMMC, about half of the participants (49%) indicated they had an increased frequency of erections or no change (26% and 23%, respectively) compared with pre-VMMC, and about one-third (34%) reported a decrease (17% no opinion). Just over half (53%) of the respondents who had undergone VMMC reported increased orgasms or no change in orgasms (33% and 20%, respectively), and one-third (33%) reported fewer orgasms (14% no opinion). Finally, about half (51%) reported increased time to achieve orgasm or no change (35% and 16%, respectively), and one-third (35%) reported less time to orgasm (14% no opinion).

**TABLE 3:** Post-Voluntary Medical Male Circumcision (Post-VMMC) Sexual Functioning and Satisfaction Among Zambian Men (N = 256)^a^ and Female Partners (N = 159)

	Men	Female Partners
**Sexual Functioning**		
Erections		
More	67 (26%)	
No change	58 (23%)	
Fewer	86 (34%)	
No opinion	45 (17%)	
Orgasms		
More	84 (33%)	
No change	50 (20%)	
Fewer	85 (33%)	
No opinion	37 (14%)	
Time to orgasm		
Increased	89 (35%)	
No change	41 (16%)	
Decreased	91 (35%)	
No opinion	35 (14%)	
**Sexual Satisfaction**		
Increased	107 (42%)	99 (63%)
No change	40 (15%)	25 (16%)
Decreased	56 (22%)	21 (13%)
No opinion	54 (21%)	14 (8%)
**Appearance**		
Better	159 (62%)	97 (61%)
No difference	52 (20%)	24 (15%)
Worse	39 (15%)	23 (15%)
No opinion	6 (3%)	15 (9%)
**Cleanliness**		
Cleaner/easier to keep clean	180 (70%)	112 (70%)
No difference	33 (13%)	22 (14%)
Less clean/more difficult to keep clean	34 (13%)	18 (11%)
No opinion	9 (4%)	7 (5%)

All data are reported as No. (%).

a 1 male participant was missing all post-VMMC data.

### Sexual Satisfaction

Domains of sexual satisfaction analyzed included intercourse satisfaction, overall satisfaction, and sexual desire. Neither the quadratic nor linear growth curve components of intercourse satisfaction were significant (*P* = .09 and .84, respectively), thus intercourse satisfaction did not change over time. Examination of overall satisfaction revealed no quadratic change in time (*P* = .42); however, overall satisfaction increased linearly over time (*P* = .007; mean, baseline = 7.09, 6 months = 7.31, 12 months = 7.53). Finally, analysis of sexual desire showed a linear increase over time (*P* = .001) with a quadratic decrease (*P* = .02); estimated mean scores were 6.25, 6.67, and 6.79 at baseline, 6 months, and 12 months (Table 2).

Following VMMC, 57% of male participants reported that sexual activity was more pleasurable or that there was no change (42% and 15%, respectively), and 22% reported decreased sexual satisfaction (21% no opinion). Female partners also indicated their level of sexual satisfaction following their partner’s VMMC; most (79%) reported increased sexual satisfaction or no change (63% and 16%, respectively), and 13% reported decreased satisfaction (8% no opinion) (Table 3).

Table 4 presents agreement between partners on post-VMMC data; there was 76% agreement between partners in post-VMMC sexual satisfaction. Men undergoing VMMC were asked to rate their overall level of satisfaction with the procedure (0 = not at all satisfied, 10 = extremely satisfied). The mean satisfaction score was 8.4 (standard deviation, 2.7), and 96% of participants (n = 245) indicated they would recommend VMMC to a friend. In addition, 94% (n = 150) of female partners reported they would recommend VMMC, based on their and their partners’ experience with the procedure.

96% of men undergoing VMMC said they would recommend the procedure to a friend.

**TABLE 4:** Agreement Between Zambian Partners on Sexual Satisfaction, Appearance, and Cleanliness Following Voluntary Medical Male Circumcision

Women	Men		Kappa (95% CI)
	Increased (or Better)/ No Change	Decreased (or Worse)	
Sexual satisfaction^a^			0.25 (0.04, 0.45)
Increased/no change	80 (68%)	18 (15%)	
Decreased	10 (9%)	9 (8%)	
Appearance^a^			0.10 (-0.09, 0.28)
Better/no change	104 (73%)	15 (11%)	
Worse	18 (13%)	5 (3%)	
Cleanliness^a^			0.20 (-0.01, 0.42)
Increased/no change	119 (80%)	11 (7%)	
Decreased	13 (9%)	5 (4%)	

All data are reported as No. (%).

Abbreviation: CI, confidence interval.

a The sample size of couples differed across items because some participants did not provide an opinion on all items. For sexual satisfaction, the sample size was 117 couples; for appearance, 142 couples; and for cleanliness, 148 couples.

### Appearance and Cleanliness

Male participants undergoing VMMC were asked about the appearance of their penis post-VMMC; most (82%) reported that it looked better or neither better nor worse (62% and 20%, respectively), and 15% reported that it looked worse (3% no opinion). Female partners reported similar feelings about the appearance of their partner’s penis; the majority (76%) reported that it looked better or that there was no difference (61% and 15%, respectively), while 15% indicated that it looked worse (9% no opinion). There was 77% agreement between partners in post-VMMC penile appearance. In addition, most (83%) male participants reported increased penile cleanliness or no change following VMMC (70% and 13%, respectively), while 13% reported that it was harder to keep clean (4% no opinion). Most (84%) female partners reported increased cleanliness of their partner’s penis following VMMC or no change (70% and 14%, respectively), and 11% indicated that the penis was less clean (4% no opinion); 64% agreement between partners. The Figure presents a graphic representation of men’s and women’s responses concerning penile appearance and cleanliness.

Most men and women reported increased penile cleanliness following VMMC.

**FIGURE.**
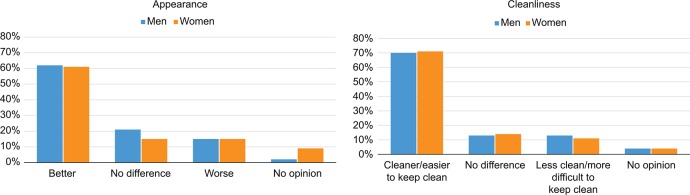
Perceptions of Penile Appearance and Cleanliness Following Voluntary Medical Male Circumcision Among Men (N=256) and Their Partners (N=159)

### Early Resumption of Sexual Intercourse

Among men undergoing VMMC, 178 (69%) reported having sexual intercourse at least once between the procedure and their 3-month post-VMMC assessment. Of those sexually active men, 135 (76%) waited at least 6 weeks before resumption of intercourse following VMMC, but 43 men (24%) did not wait.

Most men reported waiting at least 6 weeks before resuming sex following VMMC.

To investigate factors associated with early resumption of sex, men who had sex but waited at least 6 weeks were compared with those who did not wait; results are presented in Table 5. In summary, early resumption of intercourse was associated with increased HIV risk behavior (27% of high-risk participants resumed sex early vs. 10% of low-risk participants, *P* = .01) as well as with the increased risk of infection or tearing of the surgical incision; self-reported tearing of the incision was noted by 15% of participants and infection noted by 11%; 47% of participants resuming sex early described infection or tearing vs. 20% of those waiting at least 6 weeks (*P*<.001). Additionally, participants resuming sex early reported decreased orgasm functioning (*P*<.001), overall satisfaction (*P* = .001), and sexual desire at the post-VMMC visit (*P* = .05) (Table 5). Demographics and VMMC knowledge did not impact early resumption of sexual intercourse.

**TABLE 5:** Factors Associated With Early Resumption of Sex Following Voluntary Medical Male Circumcision (VMMC), by Timing of Resumption of Sex (N = 178)^a^

	Waited at Least 6 Weeks (n = 135)	Did Not Wait at Least 6 Weeks (n = 43)	t/χ^2^	*P* Value
*Demographic Characteristics*				
Age, mean (SD), years	28.3 (8.4)	26.4 (8.5)	1.3	.20
Education level, No. (%)			2.1	.15
High	103 (76%)	28 (65%)		
Low	32 (24%)	15 (35%)		
Married/cohabitating, No. (%)			1.8	.18
Yes	66 (49%)	16 (37%)		
No	69 (51%)	27 (63%)		
Wants (more) children, No. (%)			2.9	.09
Yes	64 (47%)	14 (33%)		
No	71 (53%)	29 (67%)		
**HIV risk category,**^b^ **No. (%)**			**6.7**	**.01**
** High**	**12 (10%)**	**11 (27%)**		
** Low**	**105 (90%)**	**30 (73%)**		
*Adverse Events*				
**Post-VMMC infection or tearing, No. (%)**			**11.8**	**<.001**
** Yes**	**27 (20%)**	**20 (47%)**		
** No**	**108 (80%)**	**23 (53%)**		
*Sexual Satisfaction*				
Erectile functioning, mean (SD)	17.6 (6.5)	17.7 (5.5)	0.1	.94
**Orgasm functioning, mean (SD)**	**5.8 (3.5)**	**3.3 (3.4)**	**4.1**	**<.001**
Intercourse satisfaction, mean (SD)	7.4 (2.4)	7.7 (1.7)	0.7	.48
**Overall satisfaction, mean (SD)**	**8.3 (2.2)**	**6.5 (3.2)**	**3.4**	**.001**
**Sexual desire, mean (SD)**	**6.9 (1.8)**	**6.2 (2.1)**	**2.0**	**.05**
*VMMC Knowledge*				
Correct knowledge, mean (SD)	11.8 (2.4)	11.9 (2.5)		.71^c^
Misinformation, mean (SD)	5.5 (2.2)	6.0 (2.4)		.20^c^

Abbreviation: SD, standard deviation.

a Among men reporting at least one instance of sexual intercourse between the VMMC procedure and their 3-month post-VMMC assessment.

b The sample size of men with HIV risk designation comprised only 158 men because of missing items on the sexual risk behavior questionnaire.

c Wilcoxon’s test.

Note: Statistically significant differences are noted in boldface.

## DISCUSSION

This study examined post-VMMC responses of Zambian men and their female partners. Overall, outcomes suggest VMMC was acceptable to both men and their partners.

Self-reported sexual functioning, including erectile and orgasm function, increased or was unaffected by VMMC. Overall sexual satisfaction, including satisfaction with intercourse, appeared better or unchanged among the majority of the male participants and their female partners. In fact, men and their partners expressed high levels of agreement in their assessment of sexual satisfaction following VMMC. An increase in sexual satisfaction among women was also noted in a study in Turkey by Senkul et al.^31^ that found there was a delayed ejaculatory time in those circumcised. Senkul and colleagues suggested that delayed ejaculation in those who were circumcised should be regarded as an advantage rather than a complication. In a study in Canada, Payne et al.^32^ also evaluated sexual arousal and compared circumcised and uncircumcised men, obtaining similar results.

Contrary to men’s pre-VMMC concerns that circumcision may impair sexual performance, satisfaction, and pleasure, the findings from this study suggest that both men and their partners can expect equal or increased sexual satisfaction as well as improved penile hygiene following VMMC. Having women participate in VMMC programs also provides an opportunity to convey important information concerning how VMMC can protect women’s health, given the high rates of human papillomavirus (HPV) and cervical cancer in Zambia. It is not clear whether men and women agreed on circumcision because they shared the same attitudes or because they *evolved* to share the same views. Interventions to enhance VMMC uptake that include women will also assist women in better understanding how VMMC can impact their sexual satisfaction as well as their health.^13^^,^^14^^,^^33^

Findings suggest that most men and their partners can expect equal or increased sexual satisfaction as well as improved penile hygiene post-VMMC.

Results from the Spear and Shield study^13^^,^^14^ and previous research suggest that women’s perceptions, attitudes, and opinions about VMMC may be important to men. Previous research by this team found that men were interested in knowing women’s preferences for the appearance of their penis, i.e., circumcised or uncircumcised. The majority of men felt the appearance of their penis had improved following VMMC, and there was a high level of agreement between male and female partners regarding this perception. The enhanced appearance of the penis was also noted in previous studies using the Shang Ring device in comparison with conventional VMMC methods (dorsal slit method in Zambia and forceps-guided method in Kenya). Prepex studies had similar results in comparison with surgical methods.^34^^–^^37^ Additionally, most men and women agreed that VMMC enhanced penile cleanliness; increased cleanliness may also play a role in stimulating arousal and may also be responsible for increased sexual satisfaction.

Women’s perceptions and attitudes about VMMC may be important to men.

Prolonged abstinence from sexual activity is one of the principal reasons why clients are hesitant to undergo VMMC.^6^^,^^38^ In this study, complications were associated with premature resumption of sexual activity prior to adequate healing, highlighting the need for interventions to deter early resumption of sexual activity post-VMMC.^39^ Most of the participants who resumed sex prior to the recommended 6 weeks also had high-risk histories; this group also suffered a higher rate of self-reported post-surgical complications, including increased rates of post-surgical infection and decreased sexual satisfaction, as illustrated in Table 5. Although other factors could have played a role in poor surgical outcomes, e.g., wound care, cleanliness, and other post-surgery activities, it is clear that early resumption of sexual activities is an important contributor to unfavorable outcomes, particularly for those with high-risk histories.

Several studies have shown that significant numbers of men may start sexual activity before the 6-week healing period elapses.^39^^,^^40^ This problem will increase as devices for VMMC, such as PrePex or the Shang Ring, are introduced. Wound healing after VMMC when using such devices is slower than when the conventional surgical method is used, as secondary healing, or secondary intention, is slower than primary healing, or primary intention, in which suturing is done. Although a meta-analysis by Mehta et al.^40^ indicated that early sex did not increase likelihood of HIV acquisition, the study addressed those who had VMMC using conventional surgical methods in which wounds generally heal faster than when using device-based methods.^34^^–^^37^ Thus, increased attention should be devoted to assisting post-VMMC clients with delaying resumption of sexual intercourse for at least 6 weeks, especially when devices are used.^34^^–^^37^ Efforts to deter men from early resumption of sexual intercourse, for example, educational and reminder text messaging,^40^ have thus far not been successful. Hewett et al.^41^ reported that some men resumed sex with stitches still intact. Additional research on this refractory issue is sorely needed.

### Limitations

The primary limitation of this study was the relatively small amount of data collected from women, which prevented a comprehensive evaluation of the role of VMMC in women’s sexual satisfaction. In addition, although agreement between partners regarding penile appearance and cleanliness was high, the Kappa statistics were not statistically significant. This was very likely due to the low variability in those data, which resulted in a very high probability of agreement “by chance.”^42^^,^^43^ More finely grained questions should be considered to examine agreement between men and women on these issues. Additionally, self-reported problems arising post-VMMC were high and may have been misinterpreted by participants; the accuracy of these data would have been enhanced by clinical examination. Further, sexual satisfaction was not addressed in the intervention, and no differences between control and intervention conditions were noted in sexual satisfaction at baseline or follow-up. Though there was no reason to assume that the intervention would have had any impact on sexual satisfaction, future studies could explore the potential influence of VMMC interventions on sexual satisfaction. Finally, this study used a self-selected group of men seeking HIV testing at CHCs, assuming that these men would represent a level of HIV risk that is higher than in the general population. Future studies could further examine the impact of VMMC on sexual satisfaction in the general population.

## CONCLUSION

Zambian men undergoing VMMC and their partners had a high degree of satisfaction with the procedure and its consequences in terms of sexual satisfaction, validating findings from previous research. Women’s opinions and preferences regarding VMMC may be valued by men, and scale-up of VMMC could be influenced by including women when introducing VMMC-promoting interventions. Premature resumption of sexual intercourse was associated with an increase in adverse events, underscoring the importance of further investigation to develop effective interventions to delay resumption of sex during the healing period.

## Notes

**Peer reviewed**
